# The Foamy Virus Gag Proteins: What Makes Them Different?

**DOI:** 10.3390/v5041023

**Published:** 2013-03-26

**Authors:** Erik Müllers

**Affiliations:** 1 Department of Cell and Molecular Biology, Karolinska Institutet, SE-17177 Stockholm, Sweden; 2 Institut für Virologie, Medizinische Fakultät “Carl Gustav Carus”, Technische Universität Dresden, Fetscherstr. 74, 01307 Dresden, Germany

**Keywords:** Foamy Virus Gag, functional domains, virus assembly, GR box, nuclear localization

## Abstract

Gag proteins play an important role in many stages of the retroviral replication cycle. They orchestrate viral assembly, interact with numerous host cell proteins, engage in regulation of viral gene expression, and provide the main driving force for virus intracellular trafficking and budding. Foamy Viruses (FV), also known as spumaviruses, display a number of unique features among retroviruses. Many of these features can be attributed to their Gag proteins. FV Gag proteins lack characteristic orthoretroviral domains like membrane-binding domains (M domains), the major homology region (MHR), and the hallmark Cys-His motifs. In contrast, they contain several distinct domains such as the essential Gag-Env interaction domain and the glycine and arginine rich boxes (GR boxes). Furthermore, FV Gag only undergoes limited maturation and follows an unusual pathway for nuclear translocation. This review summarizes the known FV Gag domains and motifs and their functions. In particular, it provides an overview of the unique structural and functional properties that distinguish FV Gag proteins from orthoretroviral Gag proteins.

## 1. Introduction

While the retroviral Env mediates the important steps of receptor binding and membrane fusion, and Pol provides the key enzymatic functions required for viral replication, it is the Gag protein that is the major structural component of the viral particle. Gag functions are, however, not restricted to capsid formation; retroviral Gag proteins have numerous and very complex roles in the viral life cycle. Gag, (i) is the major component involved in intracellular trafficking processes; (ii) orchestrates viral assembly and disassembly; (iii) regulates viral gene expression; (iv) mediates correct encapsidation of Pol, the viral genome, and accessory proteins; (v) is involved in spatiotemporal regulation of the essential, viral, enzymatic reactions; and (vi) is essential for viral budding [reviewed in 1].

The family of *Retroviridae* (retroviruses) is divided into two subfamilies: the *Orthoretrovirinae* (orthoretroviruses) and the *Spumaretrovirinae* (spumaretroviruses). Whereas the subfamily of orthoretroviruses consists of six genera, the subfamily of spumaretroviruses consist of only one genus—the spumaviruses, also termed Foamy Viruses (FV). FV infection is endemic in many new- and old-world monkeys from which sporadic zoonotic transmissions to humans can occur. Transmission directly between humans has never been observed [reviewed in 2]. Other natural hosts include cats (feline FV, FFV), cattle (bovine FV, BFV) and horses (equine FV, EFV) [3]. Although FVs share the major characteristics of retroviruses in that they reverse-transcribe and integrate their genome, FV replication deviates in many aspects from that of orthoretroviruses [reviewed in 4,5]. In particular the FV Gag proteins have strikingly different domains and functions compared to orthoretroviral Gag, many of which are reminiscent of another reverse transcriptase encoding virus family—the hepadnaviruses [reviewed in 6].

This review summarizes the current knowledge of the different FV Gag domains and motifs and their functions, highlighting common features, but more importantly concentrating on the differences between foamyviral and orthoretroviral Gag proteins. The following parts will focus mainly on the features of prototype FV (PFV) Gag, as PFV is the best characterized isolate and represents the model for the FV family. PFV was isolated in the early 70s from a human nasopharyngal carcinoma [7], with the isolate initially named human FV (HFV), but was later renamed to PFV when large homologies to simian FV (SFV) of chimpanzees became apparent [8,9]. If amino acid positions are specified in the text these refer to the respective position in PFV Gag, however, statements are generalized if applicable.

## 2. Functional Domains of PFV Gag and Their Role in Viral Replication

Nascent FV Gag is translated as a precursor protein on free ribosomes in the cytoplasm from the unspliced, genomic RNA. In contrast to all other retroviruses, FV only express a Gag, and not a Gag-Pol fusion protein [10,11,12]. In general, the primate FV Gag proteins are slightly larger in size ranging from 572 aa (SFV of squirrel monkey) to 653 aa (SFV of chimpanzees) compared to 559 aa, 544 aa, and 489 aa for EFV, BFV and FFV Gag respectively. PFV Gag has a size of 648 aa. In contrast to orthoretroviruses, FV Gag is not processed into matrix (MA), capsid (CA) and nucleocapsid (NC). Instead, only a limited primary proteolysis by the FV protease (PR) occurs, which leads to the removal of a small C-terminal peptide [13,14]. In the case of PFV, cleavage of the 71 kDa precursor (p71^Gag^) results in a 68 kDa (p68^Gag^) and a 3 kDa (p3^Gag^) cleavage product. FV Gag processing is essential for viral infectivity [15,16,17]. In infectious PFV particles, p71^Gag^ and p68^Gag^ are present in a ratio between 1:1 and 1:4 [4,18]. Due to its small size it is unknown if p3^Gag^ is present in released viral particles. It is also unknown if it has a specific function in the viral life cycle.

**Figure 1:**
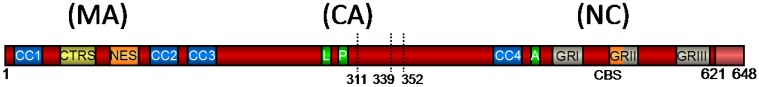
**Schematic Representation of the Prototype Foamy Virus (PFV) Gag Precursor Protein [adapted from 22].** Numbers indicate the amino acid position in PFV Gag. The primary cleavage site is indicated by its amino acid position (621). Secondary cleavage sites are indicated by dotted lines and their amino acid position. CC1 – CC4, predicted coiled-coil motifs 1–4; CTRS, cytoplasmic targeting and retention signal; NES, nuclear export signal; L, late assembly domain; P, Pro-Pro-Pro-Ile motif (PPPI motif); A, assembly domain; GRI–III, glycine-arginine boxes I–III; CBS, chromatin-binding site; MA, matrix domain; CA, capsid domain; NC, nucleocapsid domain.

In addition to the primary Gag cleavage site, three secondary cleavage sites were identified in the central Gag region at positions 311/312, 339/340, and 352/353. Processing at these sites was shown for primate and non-primate FV isolates [14,19]. Secondary processing occurs with much lower efficiency and C-terminal p68^Gag^/p3^Gag^ processing seems to be a prerequisite for further proteolysis [14]. The importance of secondary FV Gag processing has been debated. Mutations at any of the three secondary cleavage sites do not abrogate particle release, but lead to noninfectious viruses [14,20]. Interestingly, despite their close proximity, a mutation in one cannot be functionally substituted by the presence of the two other cleavage sites. This suggests that all three secondary cleavage sites have an important function. Initially it was thought that further Gag processing by Pol at secondary cleavage sites was required for capsid disassembly upon target cell entry [13,14,20]. However, we recently found that viral protease activity is fully dispensable during target cell entry [21]. Thus, the exact function of secondary Gag cleavage remains to be determined. Despite the lack of processing into mature subunits orthoretroviral-like MA, CA and NC domains were initially assigned to the N-terminal part, the central region, and the C-terminal part of FV Gag, respectively, because these different parts of FV Gag harbor similar motifs and often perform similar functionality as the orthoretroviral MA, CA, and NC subunits (Figure 1) [reviewed in 4].

Sequence homology between different retroviral Gag proteins is quite low. In line with that, Gag proteins of different foamyviral isolates have on average of less than 50% amino acid similarity. However, as all retroviral Gag proteins perform similar functions to achieve viral replication, similar functional domains are present in most retroviral Gag proteins (Figure 1).

### 2.1. FV Gag Domains Found Also in Orthoretroviruses

To begin our overview of the FV Gag domains we shall briefly review common Gag domains, which FV Gag shares with some of its orthoretroviral counterparts. These are the domains for incorporation of the envelope protein (Env), the cytoplasmic targeting and retention signal (CTRS) that directs Gag to the region where particle assembly occurs, interaction (I) domains that are essential for Gag-Gag interactions during capsid assembly, and late (L) domains that are required for particle release [23].

Several studies show that the N terminus of FV Gag is essential for the interaction of Gag with Env [18,24,25,26]. Thereby, the structural integrity of a predicted coiled-coil motif (CC1) at aa 4*–*19 seems to be important for the interaction [18]. Other retroviral Gag proteins, like HIV-1 Gag, contain similar domains required for efficient Env incorporation in the Gag N terminus [1]. However, FV are unique among retroviruses as FV Gag lacks a membrane-targeting domain (see below). Therefore, viral budding requires the co-expression and interaction of Gag and Env [27,28]. This feature is most closely resembled by Mason Pfizer Monkey Virus (MPMV), where although Env co-expression is not required, it was shown to enhance viral particle release by mediating capsid transport [29]. Similarly, it is thought that FV Env interacts with the preassembled capsids at a pericentriolar region, probably the trans-Golgi network, which then leads to membrane targeting of the fully assembled viral particles [30].

Like for B/D type orthoretroviruses, FV Gag preassembles into capsid structures in the cytoplasm at the centrosome [18,30,31]. Targeting of assembly is mediated by a CTRS at aa 43–60. The CTRS comprises of a [GXWGX_3_RX_7_L(Q/V)D] motif centered around an essential arginine residue, which is conserved among all known FV Gag proteins [32]. The presence of a CTRS in the Gag N terminus is reminiscent of MPMV, which also uses a B/D‑type egress strategy [33].

Viral I domains are essential for capsid formation, as they mediate Gag-Gag interaction and thereby Gag multimerization. They contain high amounts of basic amino acids and can be functionally substituted between different retroviruses. The I domains function to provide the proper density to viral particles. It is thought that they influence particle density by interacting with nucleic acids and using them as a scaffold to facilitate the subsequent Gag-Gag protein interactions [reviewed in 34]. Most orthoretroviral I domains are found in the C-terminal part of the CA subunit and in the NC subunit. Similarly, FV Gag contains an I domain in the very C-terminal part of the central region. The CC2, found at aa 130–150 was shown to be essential for Gag-Gag interactions [35]. Additional regions with putative I domain function can be found in the C-terminal part of FV Gag, the respective NC domain (also see Figure 1). At aa 464–469 a Tyr-X-X-Leu-Gly-Leu motif (YXXLGL motif) with important functions for capsid assembly was found. According to its function the YXXLGL motif was termed ‘assembly domain’ [35,36,37]. The assembly domain is well conserved among all FV species and was attributed to I domain function [37]. In close proximity to the assembly motif lies glycine and arginine rich box (GR box) I (GRI) (aa 485 – 512), which is also required for correct capsid assembly [38]. Notably, orthoretroviral I domains often overlap with the Cys-His motifs [36] and the FV GR boxes are the functional equivalent of the Cys-His motifs (also see below) [38,39]. While according to the original definition, GRI is not an I domain, because particles with mutations in GRI appear at the same buoyant density as wild-type particles [38], Cimarelli and Luban showed for HIV‑1 Gag that the determinants of the NC I domain and of virion density are genetically distinguishable [40]. Thus, it is possible that the PFV Gag region comprising the YXXLGL motif as well as GRI together form a larger I domain with functions in particle assembly and particle morphology, but not for particle density.

The FV Gag assembly motif was initially discovered together with two additional motifs, when sequences that show homologies to known L domains were analyzed [41,42]. L domains function during the last step of the viral replication cycle. They mediate interaction with components of the vacuolar protein sorting (VPS) machinery for particle release [reviewed in 43]. L domains are found in different domains of the retroviral Gag and some Gag proteins contain several L domains. While the L domains of lentiviruses are located mainly in the C terminus within Gag, those of oncoretroviruses and FVs are often found in the central region and the N-terminal half of the Gag precursor [reviewed in 43]. L domains act independently of their respective localization in Gag and can be functionally substituted between different retroviruses [reviewed in 44,45]. PFV Gag contains a Pro-Ser-Ala-Pro motif (PSAP motif) with L domain function at aa 284*–*287 [41,42]. All known SFV Gag proteins except SFV of orangutan (SFVora) Gag contain PSAP motifs in the central region. In addition, SFVora Gag and also SFV of spider monkeys contain a PSAP motif in the very C terminus [41,46]. The isolates of bovine, equine, and feline FV lack both PSAP and also Pro-Pro-X-Tyr sequence motifs. They do, however, contain several Leu-X-X-Leu motifs, but these are not well conserved [41]. As the conserved YXXLGL motif has no classical L domain function (see above) it is currently unknown if and how these FV isolates utilize the VPS for particle release.

An additional well-conserved motif in primate FV Gag that was initially thought to have L domain function is a Pro-Pro-Pro-Ile motif (PPPI motif) at aa 298*–*301. This motif clearly is not an L domain, as it was found to be dispensable for particle release. However, mutant particles have strongly diminished infectivity [37,41,42].

### 2.2. FV Gag Proteins Lack Several Characteristic Orthoretroviral Domains

Analysis of the first foamyviral Gag sequence, the sequence of PFV Gag, [47] highlighted several peculiarities of FV Gag. This includes the apparent lack of several hallmarks of orthoretroviral Gag proteins such as the membrane-binding (M) domain, the major homology region (MHR) and the zinc-finger motifs (Cys-His motifs), but also the rare appearance of lysine residues [48].

M domains are required for transport and binding of Gag to the plasma membrane. In most orthoretroviruses the N-terminal glycine residue of the MA subunit is myristoylated. The myristyl-residue and the N-terminal basic amino acids mediate Gag transport and binding to the plasma membrane. FV Gag does not contain an M domain and is therefore not targeted to the plasma membrane [49]. Instead, membrane-targeting of the pre-assembled capsids is mediated through interaction with the FV Env protein (see above) [reviewed in 50]. By adding an artificial myristoylation signal, FV Gag can be targeted to the plasma membrane and virus-like particles (VLP) are released in an orthoretroviral-like manner [25,32,51,52,53]. However, the released virions show strongly decreased or undetectable infectivity [reviewed in 53].

There is one highly conserved sequence in the Gag of orthoretroviruses—the MHR in the CA subunit. The MHR includes a stretch of approximately 20 residues that are well conserved even among unrelated retroviruses, which otherwise show little sequence homology in CA [54]. The MHR is involved in virus assembly; however, its exact function is unknown. The MHR lacks FVs [4], and as the function of the orthoretroviral MHR is still debated it remains unclear if there is a similar domain in FV Gag.

The orthoretroviral NC subunit contains a high number of basic residues and, most notably, one or two copies of a conserved motif composed of regularly spaced cysteine and histidine residues, the Cys-His motif. Cys-His motifs are essential for orthoretroviral replication [55,56]. They are required for RNA recognition and packaging, particle assembly and release, and reverse transcription [reviewed in 57,58]. In contrast to orthoretroviruses, FV Gag lacks the Cys-His motifs. Instead, all FV Gag proteins contain a high percentage of glycine and arginine residues in the C-terminal region [31,59]. Interestingly, the structure of FV Gag resembles that of Gag of the *Drosophila* retrovirus Gypsy, which is also not processed to MA, CA, and NC subunits and lacks the hallmark orthoretroviral Cys-His motifs [60].

### 2.3. The FV Gag GR Boxes: A Functional Equivalent of Orthoretroviral Cys-His Motifs

The Cys-His motifs are highly conserved throughout the subfamily of orthoretroviruses and they are essential for several steps of orthoretroviral replication. However, despite their importance these motifs are not found in FV Gag. The following section, therefore, focuses on the GR-rich C terminus of FV Gag and how it mediates similar functions to the Cys-His motifs.

**Figure 2:**
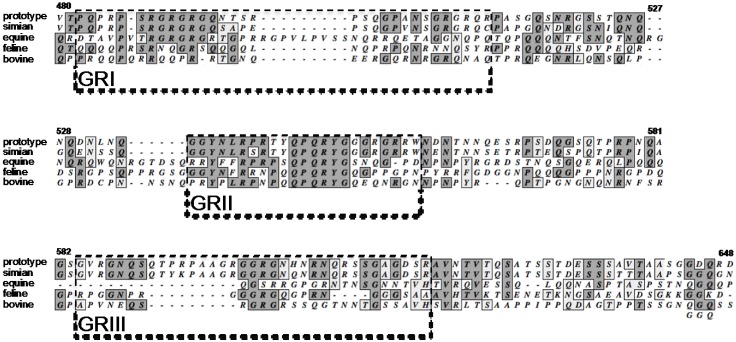
**Sequence Alignment of the C-terminal Region of Different FV Species’ Gag.** Sequences were obtained from the NCBI GenBank database: prototype, prototype foamy virus (NC_001736); simian, simian foamy virus of chimpanzees (NC_001364); equine, equine foamy virus (NC_002201); feline, feline foamy virus (NC_001871); bovine, bovine foamy virus (NC_001831). Sequences were aligned using MacVector (MacVector) software and a Gonnet matrix. Sequence identities are shaded dark. Sequence similarities are shaded light. Numbers indicate amino acid position in PFV Gag. The boxed amino acids indicate the PFV Gag glycine and arginine rich boxes (GR boxes) I–III as designated by Schliephake *et al.* [61].

The C terminus of all FV Gag proteins contains a high number of glycine and arginine residues and in the different SFV Gag proteins and in PFV Gag, the residues are clustered in three GR-rich boxes (GR boxes) [46,61]. While no GR boxes were assigned for the non-primate FV Gag proteins, the amino acid sequence, especially of GRII and GRIII, is well conserved throughout all FV Gag proteins (Figure 2) [3,31,59,62].

The PFV GR boxes have long been implicated in RNA encapsidation and Pol packaging. In an early study, all three GR boxes were found to bind nucleic acids *in vitro* and the FV Gag C terminus was implicated in RNA binding [39]. The structure of FV Gag is therefore similar to that of the core protein of hepadnaviruses, which also binds RNA or DNA through specific arginine residues in the C terminus [63]. Later studies strengthened the view that the FV Gag GR boxes are required for viral RNA binding [64]. Furthermore, it became apparent that they also mediate Pol packaging. It is thought that Gag, via the GR region, as well as the Pol precursor protein, both bind to (pre)genomic RNA, where the RNA serves as a bridging molecule [39,64,65,66]. This mechanism would elegantly connect RNA encapsidation and Pol packaging. However, this concept was somewhat contradicted by the finding that different GR box-deletion mutants package RNA in amounts similar to those of the wild-type virus [38,67]. Possibly the different results originate from the use of different viral expression systems, different length of the introduced mutations, and different mutational approaches (deletion, substitution, truncation). Lee and Linial convincingly showed that the presence of a few basic residues is sufficient for GRI box function [67]. Additionally, we found that substituting the GR boxes has more severe effects than deletions, whereby the size of the introduced substitution correlates with the adverse effects observed [38]. Therefore, it seems apparent that the FV Gag C terminus mediates its function not through specific single amino acids, but it is rather the overall structure and charge that determines nucleic acid binding [38,67]. This suggests that RNA encapsidation might depend on the relative amount of charged residues in the FV Gag C terminus.

**Figure 3:**
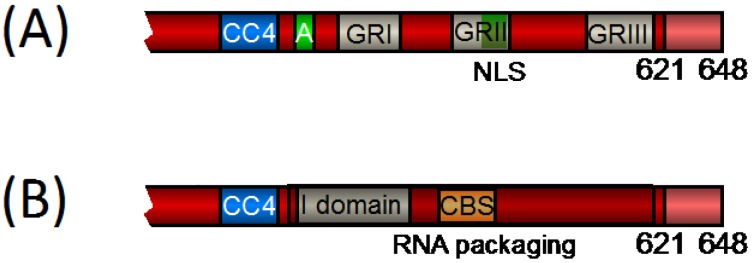
**Schematic Representation of the C-terminal Domain of PFV Gag**. (**A**) Domain structure of the PFV Gag C terminus as previously designated. (**B**) Our model of the PFV Gag C terminus, integrating the recent available data. Numbers indicate the amino acid position in PFV Gag. The primary cleavage site is indicated by its amino acid position (621). CC4, predicted coiled-coil motif 4; A, assembly domain; GRI–III, glycine-arginine-rich boxes I–III; NLS, nuclear localization signal; CBS, chromatin-binding site; I domain, interaction domain. The shaded region indicates the glycine and arginine-rich C terminus required for RNA packaging.

Based on the current results one might want to rethink the concept of clustered PFV GR boxes as originally designated by Schliephake *et al.* [61]. Clearly the arginine residues in the C terminus of all FV Gag proteins have prominent functions in the viral life cycle. However, it seems not to be the boxed motifs but rather the overall charge that is important for RNA packaging [67] (Figure 3). Furthermore, newly available protein sequence data for other FV species shows that the sequences flanking the actual GR boxes are equally well conserved as the GR box regions (also see Figure 2). Therefore, ‘non-GR box’ sequences in the Gag C terminus might call for equal research consideration to elucidate if and how the FV Gag GR-rich C terminus couples RNA encapsidation and Pol packaging.

In addition to their functions for RNA encapsidation and Pol packaging, all three PFV GR boxes are also required for correct reverse transcription of the viral RNA. It has previously been shown for FV and other retroviruses that correct reverse transcription and capsid morphology are two tightly linked functions as even slight defects in retroviral capsid assembly can severely affect reverse transcription [37,55]. This correlation holds only partially true for the GR boxes. While we found that the integrity of GRI is important for both correct capsid morphology and reverse transcription, mutations in GRII or GRIII led to severely impaired reverse transcription without obvious defects in particle morphology [38].

In summary, the FV Gag GR motifs appear as a true functional equivalent to the orthoretroviral Cys-His motifs. However, while there is clear functional overlap, there are also some functional differences. Firstly, early studies in HIV-1 showed that point mutations that disrupt both Cys-His motifs significantly reduce particle production [55]. Furthermore, in the case of murine leukemia virus (MLV) and Rous sarcoma virus (RSV), Gag NC is strictly required for particle production [68,69]. For PFV, the Gag C terminus is dispensable for the assembly and release of viral particles [18]. Secondly, GRII harbors a motif so far unique among retroviruses*—*the chromatin binding signal (CBS), which mediates nuclear translocation (see below).

### 2.4. Many Ways Lead to the Nucleus—FV Gag Nuclear Trafficking

FV Gag nuclear localization has long been known as a characteristic feature of FV infection [70]. In fact, the nuclear accumulation of the FV Gag protein is a diagnostic feature of FV in cell culture and previously it was regarded as a unique feature of FVs compared to orthoretroviruses [61,70]. However, nucleo-cytoplasmic shuttling was later shown for the Gag proteins of several retroviruses including HIV-1, MLV, MMTV, and RSV [61,71,72,73,74].

The Gag proteins of PFV, simian FV of macaques, EFV, and BFV were all shown to transiently traffic through the nucleus [3,31,62,75], while a lack of nuclear localization was reported only for FFV Gag [76]. As GRII, and especially its CBS subdomain, are better conserved in FFV Gag than in EFV or BFV Gag (Figure 2), the lack of nuclear localization of FFV Gag is most probably not caused by poor sequence conservation. While nuclear localization seems to be a common property of retroviral Gag proteins the mechanism of FV Gag nuclear translocation is unique. Initially, PFV GRII was reported to harbor a nuclear localization signal (NLS) in its C-terminal region responsible for transient nuclear targeting of PFV Gag during certain stages of the viral replication cycle [39,61]. However, recently, it has been highlighted that PFV Gag is not imported into the interphase cell nucleus and it does not contain a functional NLS. Gag nuclear translocation occurs by tethering of Gag via its CBS to accessible chromatin during mitosis [77,78]. This mechanism occurs for endogenously expressed Gag in the infected cell, as well as for Gag delivered by infecting viral particles [77,79]. Gag-chromatin association is mediated by binding of the PFV Gag CBS to H2A/H2B core histones [78].

Also very recently, a leptomycin B (LMB)-sensitive nuclear export sequence (NES) was identified in the N terminus of PFV Gag, at amino acid positions 95*–*112 [80]. An NES-defective Gag G110V mutant was retained in the cell nucleus and prevented viral particle release in a dominant-negative manner. Interestingly, while substitution of the PFV Gag NES with that of HIV-1 Rev led to nucleocytoplasmic redistribution of Gag, viral particle release and viral infectivity could not be restored [80]. Currently, it remains unclear how the CBS and the NES function are regulated during the viral life cycle.

What is the functional relevance of transient Gag nuclear targeting? PFV Gag nuclear localization is thought to occur at least twice during the viral replication cycle, once early as part of the incoming pre-integration complex (PIC), and once late when nascent Gag is expressed and viral capsids are assembled. Therefore, there are two basic hypotheses, which are not mutually exclusive*—*either Gag nuclear targeting is required during virus entry, or during virus assembly (also see Table 1).

In the case of virus entry it has been speculated that the CBS in GRII has a role in tethering the viral genome to the host cell chromatin in order to facilitate integration [78]. Hence, the dependency on cell division for Gag to enter the nucleus might explain why the FV genome is only integrated in dividing cells [81]. However, recent studies suggest that the differential cell cycle dependence of retroviral infection is not determined by nuclear targeting signals of the viral proteins and that nuclear import is not the limiting step for infection of non-dividing cells [reviewed in 82]. Interestingly, the primate FV genome can undergo intracellular retrotransposition, while FFV does not display efficient intracellular retrotransposition [83,84]. As FFV Gag is also the only foamyviral Gag protein that was not found to enter the nucleus, Gag nuclear translocation might be linked to efficient intracellular retrotransposition.

There are several potential roles for Gag nuclear targeting during late stages of the viral life cycle. For RSV, initial Gag-Gag dimerization occurs in the cell nucleus [85]. RSV Gag nuclear trafficking is important for viral RNA export from the nucleus and for efficient incorporation of vRNA [reviewed in 86]. Thus, RSV Gag oligomerization is linked to Gag-RNA binding, both occurring in the cell nucleus. Morozov and colleagues suggested a similar model for FVs, where initial complex formation of Gag, Pol, and RNA would take place in the nucleus [87]. In line with this, Renault *et al.* recently reported PFV Gag proteins to interact in the nucleus and proposed a model where Gag would be required for nuclear export of the unspliced viral RNA [80]. However, we found that Gag nuclear localization is not essential for genomic RNA encapsidation [38]. Further contradicting the proposed model, Bodem *et al.* recently reported that FV RNA is exported from the nucleus independently of viral proteins [88]. Deletion of the entire GRII significantly reduces Pol packaging [38,64], but detailed analysis of GRII showed that Pol packaging could be distinguished from Gag nuclear localization [77]. Also for Gag-Gag interactions the picture is unclear. While PFV Gag-Gag interactions were demonstrated in the nucleus [35,80], Gag mutants lacking nuclear localization do not display severe defects in viral assembly [38]. Thus, the concept of Gag nuclear translocation being required for the formation of a Gag-Pol-RNA complex remains highly debated. Other functions for retroviral Gag proteins in the cell nucleus could include transcriptional regulation of viral or cellular genes.

**Table 1:** **Overview of Potential Functions of Retroviral Gag Proteins in the Cell Nucleus.** A question mark indicates that a function has been proposed, but not conclusively shown for the respective Gag protein, or that there are contradicting reports on the subject.

	Function	Possible Examples
**Viral entry**	Nuclear translocation of the PIC	HIV‑1 (?), PFV (?)
	Integration target site selection	MLV, HIV‑1, PFV (?)
	Chromatin tethering	PFV (?)
**Transcriptional regulation**	Transcriptional enhancement of early gene expression	HIV‑1
	Regulation of cellular gene expression	(?)
**Viral assembly**	Gag dimerization	RSV, PFV (?)
	RNA binding/export	MLV, FIV, RSV, PFV (?)
	Pol binding/packaging	PFV (?)
**Other**	Binding to translation machinery	MMTV
	Intracellular retrotransposition	PFV (?)
	Regulation of splicing	MLV

In summary, while several retroviral Gag proteins translocate to the cell nucleus, the exact functions for most of them remain unknown. Similarly, the function of FV Gag nuclear trafficking is controversially discussed. However, it is tempting to speculate that the unique mechanism of FV Gag nuclear translocation might also suggest a different functionality compared to nuclear localization of other retroviral Gag proteins.

## 3. Interaction Between FV Gag and Host Cell Proteins

As illustrated above, the Gag proteins have numerous functions in the retroviral replication cycle. To accomplish these functions, retroviral Gag proteins are highly reliant on host cell proteins. Interactions between Gag and host cell proteins are required to employ the cellular machinery for viral replication. Consequently, numerous protein interaction partners are reported for orthoretroviral Gag proteins, in particular for HIV-1 Gag [89,90].

Unlike the long list of HIV-1 Gag interacting partners, very little is known about FV Gag intracellular interactions. Given the many unconventional features of their replication, it is expected that FV Gag interaction partners await identification. However, so far only few cellular proteins have been reported to interact with foamyviral Gag proteins (Table 2).

**Table 2:** **Described Interactors of FV Gag Proteins.** The table contains both, confirmed and predicted/putative interactors. Functions are indicated as proposed in the original research articles. ‘Assay’ indicates the method by which an interaction was found. The column indicates ‘*not shown*’ if the interaction is not confirmed. Y2H, yeast two-hybrid.

Interactor	Function	Interaction Domain	Assay
**Trim5α**	Restriction of retroviral replication	CA domain	Trim5α restriction assay
**Actin**	Regulation of secondary Gag cleavage	*Unknown*	Co-immunoprecipitation
**Dynein** **LC8**	Incoming capsid transport	CC3	Co-immunoprecipitation, domain mutation
**H2A/H2B**	Chromatin tethering	CBS	Co-immunoprecipitation, domain mutation
**CRM1**	Nuclear export	NES	*Not shown*
**DDX6**	RNA encapsidation	*Unknown*	Co-localization
**TSG101**	Viral budding	PSAP L domain	Y2H

An ‘interaction partner’ that FV Gag proteins share with orthoretroviral Gag proteins is Trim5α. Trim5α is a restriction factor of the innate immune system, acting on the CA domain of Gag [reviewed in 91]. Trim5α proteins can arrest replication of several retroviruses, including equine infectious anemia virus (EIAV), MLV, HIV-1, and FV [reviewed in 92,93]. Apart from the innate immune response, an early report showed reciprocal immunoprecipitation of PFV proteins, in particular Gag, and actin. The interaction is thought to occur during viral entry. Furthermore, the authors found indications that association of Gag with actin prevents recognition of the secondary Gag cleavage sites [13]. Also during viral entry, PFV Gag interacts with the dynein light chain 8 (LC8). Petit and colleagues showed that the interaction requires the integrity of the third coiled-coil motif (CC3) at aa 160–180. Helix-breaking point mutations in this region abolish the trafficking of incoming viral capsids to the centrosome [94]. Interestingly, trafficking of different incoming retroviruses involves a centriolar step [reviewed in 95].

FV Gag from incoming capsids, as well as newly synthesized Gag translocates to the cell nucleus by interaction of the CBS with H2A/H2B core histones (see above) [77,78]. Consequently, H2A/H2B readily co-immunoprecipitates with Gag [78]. While FV Gag enters the cell nucleus by interaction with core histones, nuclear export of Gag is reported to rely on a leucine-rich, LMB-sensitive NES [80]. Leucine-rich NES are recognized by exportin 1, also named CRM1 [reviewed in 96]. Interestingly, Bodem *et al.* found that nuclear export of unspliced FV mRNA is CRM1 dependent [88]. However, FV Gag is not required for the nuclear export of FV mRNA (see above) [88] and direct interaction between Gag and CRM1 has not yet been demonstrated. Recently, the RNA helicase DDX6 was found to relocate and co-localize with viral RNA and Gag at the viral assembly site. DDX6 knockdown led to decreased RNA packaging. However, co-immunoprecipitation experiments failed to confirm a stable interaction between PFV Gag and DDX6 [97]. Finally, primate FV Gag contains classical PSAP L domains, which mediate viral budding through binding to components of the ESCRT-I complex (endosomal sorting complex required for transport), namely Tsg101 (tumor-susceptibility gene product 101) (see above) [41,42].

Based on the analogy with HIV-1 Gag, which reportedly binds to more than 50 different human proteins [89,90], it can be expected that the list of known FV Gag interaction partners is far from complete. New interaction partners and the respective interaction domains will likely be identified in the future. One possible candidate might be the fourth predicted coiled-coil motif (CC4) at aa 433–454. As CC1 is important for Gag-Env interaction, CC2 mediates Gag-Gag interactions, and the CC3 interacts with LC8, it is tempting to speculate that also CC4 might be a protein interaction domain.

## 4. Conclusions

In his review on HIV-1 Gag in 1998 Eric Freed concluded with: “A large number of studies […] have contributed significantly to our knowledge of how retroviral Gag proteins function in virus replication. Despite this wealth of knowledge, many questions remain unanswered.” [1]. Now, highlighting the similarities, but more importantly the unique features that distinguish FV Gag proteins from orthoretroviral Gag proteins leads to a very similar conclusion. Despite apparent progress on FV Gag biology, central questions are far from answered. That is, where do Gag and Env interact? How is RNA encapsidation and Pol packaging accomplished? Why does Gag localize to the cell nucleus? What is the functional role of p3^Gag^? And which host cell factors interact with Gag? Some of these questions are on the brink of being answered; some will stick around for some time to come, and new ones are certain to arise along the way. But most importantly, these are not just interesting scientific questions. The lack of an associated pathogenicity and the preferable integration pattern has led to a recent success of FV vectors in gene therapy [reviewed in 22,98,99]. However, it is clear, in order to further develop FV-derived vectors as a tool for modern molecular medicine more work is required to understand the basic molecular biology of these unconventional retroviruses.
